# Effect of a semirigid ankle brace on the in vivo kinematics and muscle activity of patients with functional ankle instability during simulated ankle sprain

**DOI:** 10.1097/MD.0000000000037832

**Published:** 2024-08-09

**Authors:** Gonghao Zhang, Chaochao Zha, Shengxuan Cao, Li Xiong, Ping Huang, Guoning Zhang, Yunhan Ji

**Affiliations:** a Department of Orthopedics, Tongren Hospital Shanghai Jiao Tong University School of Medicine, Shanghai, China; b Department of Anesthesiology, Huashan Hospital, Fudan University, Shanghai, China; c Department of Orthopedics, Huashan Hospital, Fudan University, Shanghai, China; d Shanghai Institute of Traumatology and Orthopaedics, Ruijin Hospital, Shanghai, China.

**Keywords:** brace, functional ankle instability, kinematics, simulated ankle sprain, surface electromyography

## Abstract

**Introduction:**

Ankle braces can effectively decrease the incidence of recurrent ankle sprain; however, whether the brace can decrease the severity of sprain and its related mechanism during sprain remain unknown.

**Methods:**

Twenty-two patients with functional ankle instability (FAI) (12 males and 10 females) and 16 healthy subjects (8 males and 8 females) were enrolled in this study. All of the subjects walked on a custom-built tilting platform that offered a 30° inversion (IV) to mimic the IV of ankle sprain. We collected the kinematic and surface electromyography data of patients with FAI with or without ankle brace and normal controls 6 times.

**Results:**

The FAI without brace group showed significantly higher maximum IV angles and average IV velocities than the control group (*P* < .001). The FAI with brace group revealed significantly lower maximum IV angles and average IV velocities than the FAI without brace group (*P* < .001); this group also showed significantly higher maximum external rotation (ER) angle and average ER velocities than the FAI with brace (*P* < .001) and control (*P* < .001) groups. The FAI with brace group indicated significantly lower average EMG_Prep_ (*P* = .047), EMG_Tilt_ (*P* = .037), and EMG_afterTilt_ (*P* = .004) of the peroneus longus than the FAI without brace group.

**Conclusions:**

The ankle brace could effectively decrease IV angles and their velocities and increase ER angles and their corresponding velocities during ankle sprain in patients with FAI. It could also decrease the activity of the peroneus longus muscle during ankle sprain.

## 1. Introduction

Numerous studies have focused on the mechanism of ankle sprain, recurrent ankle sprain, and subsequent functional ankle instability (FAI).^[1–4]^ Evidence reveals that ankle sprain is directly related to ankle inversion (IV) combined with extreme internal rotation but not ankle dorsiflexion (DF) or plantarflexion (PF).^[2,3]^ When the external load exceeds the self-control of ankle joints, lateral ligament injuries may occur.^[4]^ Restriction of above-ankle rotation, such as by application of an external mechanical support brace, is widely believed to decrease the incidence of ankle sprain. However, the specific kinematics and neuromuscular control of the ankle joint complex when sprain is still unclear. Naturally, we could probably not know the effect and mechanism of external mechanical support like brace during the sprain.

Induced ankle IV is a good solution to simulate the ankle sprain. Several studies have assessed kinematics and neuromuscular control during artificially induced ankle IV.^[5–9]^ Based on these studies, some researchers believe that humans can resist excessive ankle IV through the related neuromuscular control. The findings in these experiments have prompted researchers to consider the influence of the self-protection mechanism of subjects during simulated ankle sprain studies. Furthermore, most patients with FAI are afraid to walk on uneven ground, thus indicating that these patients have a degree of ego defense at the central level.^[10]^

However, there were several defects of those studies. Most reports are based on standing rapidly induced ankle IV, which cannot simulate the real conditions surrounding sprain. Moreover, whole body movement during ankle sprain cannot be studied.^[6]^ A number of scholars have conducted research featuring improved experimental conditions in which ankle sprain is simulated under more realistic conditions, such as walking or landing.^[7–9]^ However, in these studies, the subjects knew that either the trapdoor will definitely tilt and ankle IV would be induced,^[7,8]^ which resulted in a more cautious gait of subjects. Thus, accurate examination of the neuromuscular control and lower extremity kinematics during replicated LAS situations can be confounded by anticipatory responses. In order to circumvent this limitation, researchers have made improvements to create unexpected ankle sprains, such as controlling the timing of platform tilting using a trapdoor by the researcher^[11]^ or creating visual obstacles for the participants.^[12]^ Moreover, most current simulative ankle sprain studies do not measure kinematics synchronously; indeed, only 1 recent study has considered the kinematics of FAI.^[9]^ In this work, the authors found that the ankle joint IV angle and velocity at the ground contact phase are significantly lower in the expected condition than in the unexpected condition.

A brace is one of the most common ways to treat FAI and has been proven to decrease the recurrence rate of ankle sprain effectively. The hypothesis of this study is wearing a semirigid ankle brace can reduce the degree of injury during an ankle sprain by altering the kinematics and neuromuscular control of the ankle joint complex. This hypothesis aims to investigate the brace’s potential protective effect and elucidate its mechanism during an ankle sprain. The purpose of this study is to induce ankle IV suddenly under unexpected conditions among subjects with and without FAI. By measuring kinematic and neuromuscular control data synchronously before and after wearing a semirigid ankle brace, we aim to assess the brace’s impact on ankle joint stability and injury prevention. This approach will provide valuable insights into the mechanisms underlying ankle sprains and the efficacy of braces in reducing sprain severity. Ultimately, this research may inform more effective treatment and prevention strategies for ankle sprains and FAI.

## 2. Methods

### 2.1. Subjects characteristics

A total of 22 patients with FAI (12 males and 10 females, 22 feet) as FAI group and 16 subjects without FAI (8 males and 8 females, 12 feet) as control group were recruited (Table 1). For the FAI group, all of the following inclusion criteria had to be met^[13]^: (1) aged 18–40 years and body mass index between 17 to 25; (2) a history of at least 1 ankle sprain that resulted in pain, swelling, and stiffness, prohibiting participation in sports and recreational or other activities for at least 3 weeks; (3) recurrent ankle sprain (2 or more sprains in the same ankle) or giving way (more than 2 times in the past 6 months) or feeling of instability during daily life activities in the previously injured ankle; and (4) Cumberland ankle instability tool scores lower than 24. The control group had no history of ankle sprain in the last 2 years and a Cumberland ankle instability tool scores no <24. Overall exclusion criteria were a history of fracture or surgery in lower extremities, lower limb pain irrelated to ankle sprain, an ankle sprain in last 3 months, positive talar tilt test or anterior drawer test findings, and equilibrium deficits. All subjects were screened carefully to ensure that they satisfied the inclusion and exclusion criteria. This descriptive laboratory study was approved by the Institutional Review Board of Tongren Hospital Shanghai Jiao Tong University School of Medicine, and informed consent was collected from all subjects prior to their participation in this research.

**Table 1:** Subject characteristics.

	Control	FAI
Number of subjects	16	22
Number of feet	16	22
Gender	8M/8F	12M/10F
Age (years)	23.2 (1.8)	22.4 (1.6)
BMI (kg/m^2^)	20.9 (1.4)	21.6 (1.7)
CAIT scores	28.7 (0.9)	16.6 (4.1)

BMI = body mass index, CAIT = Cumberland ankle instability tool.

### 2.2. Custom-built tilting platform setup

**Figure 1.**
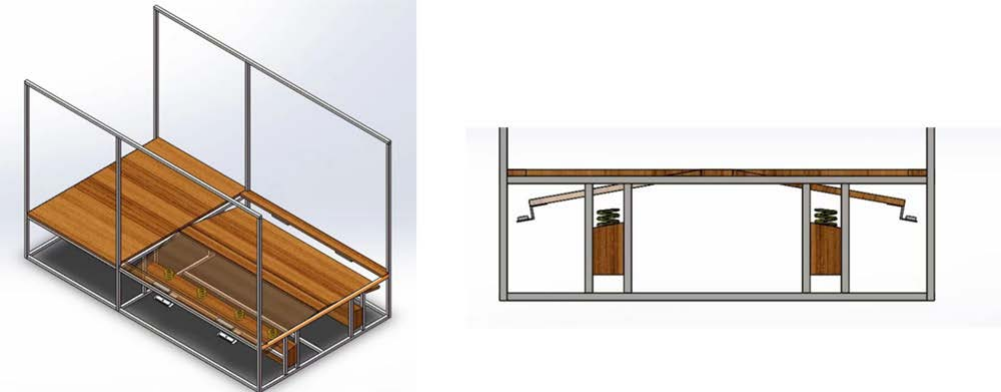
Custom-built tilting platform.

We independently designed a custom-built titling platform that included a wooden testing platform, a bilateral trapdoor with wireless electromagnetic switch, and a matched high-speed camera (Fig. 1). The platform had enough height and width for the trapdoor and a total length of over 5 m for at least 2 entire gait cycles. The trapdoor was controlled by a wireless electromagnetic switch and induced an IV deflection of 30°. A high-speed camera was placed besides the platform to divide the gait cycle. A trigger control device connected the upstream high-speed camera and the downstream wireless electromagnetic switch. A computer program ensured that tilts occur as soon as the heel-strike occurred.

### 2.3. Data process

Kinematic data were collected by an optical motion capture system including 10 cameras (Vicon Motion Systems, Oxford Metrics Ltd., Oxford, UK) with a collection rate of 100 Hz. Sixteen 14 mm retro-reflective markers, which enabled assessment of ankle joint angles, were placed on the bilateral lower extremity of the subjects.^[14]^ Marker trajectories were low-pass filtered with the fourth order zero-lag Butterworth filter at 15 Hz. Ankle joint angles were derived using the Joint Coordinate System approach, resulting in plantar/DF, IV/eversion (EV), and internal/external rotation (ER).^[15]^ The static stance served as the basis of the definition of neutral joint alignment.

The electromyography (EMG) signals of the tibialis anterior muscle (TA), the peroneus longus muscle (PL), and the soleus muscle were recorded using wireless surface electromyography (sEMG) at 2000 Hz.^[16]^ The skin was prepared to ensure an interelectrode impedance of <5 kΩ prior to data collection. The raw EMG data were filtered (10 to 750 Hz, fourth order Butterworth filter), rectified, and integrated at 80 to 250 Hz. The following time intervals were divided by the high-speed camera as follows: 100 ms before platform tilting (EMG_Prep_), during platform tilting (EMG_Tilt_), and 100 ms after platform tilting (EMG_afterTilt_).

**Figure 2.**
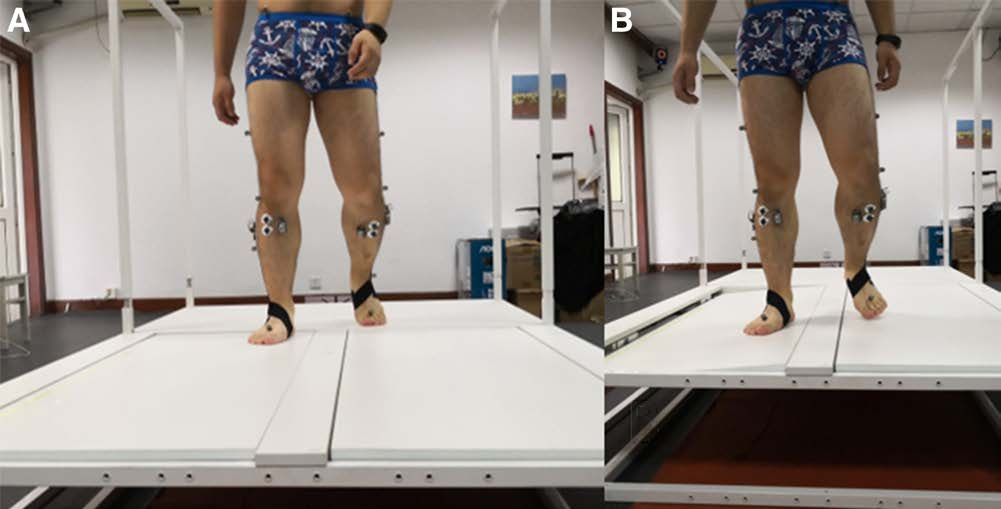
A valid collection (A. The collection starts; B. platform title).

Each subject walked on the platform with normal speed (average = 1.2 m/s), and kinematic and sEMG data, called a valid collection, were collected synchronously (Fig. 2). Data from patients with FAI were collected with or without a brace, while those from the control group were collected without the brace. Six valid collections were conducted for each condition of each foot. The tilt sides of the 6 collections were generated randomly by a computer and contained at least 3 scheming sides. The subjects and researchers were double-blinded to the collections, and all subjects were trained to walk on the platform prior to data collection.

The semirigid ankle brace (Aircast A60 Ankle Support, DJO, Europe) used in our current study was composed of nylon supporters and polyethylene lace and designed to resist IV/EV and internal/ER movements while allowing DF/plantar flexion.

In this study, we opted for the widely utilized Aircast A60 (DJO, Europe) semirigid ankle brace, which features a lace-up design. Lace-up ankle braces are a type of brace commonly worn prophylactically in athletics due to their ability to prevent IV ankle sprains. The brace has 2 crossing straps and a nylon buckle strap. Previous studies showed lace-up braces prevent IV sprains, provide proprioceptive feedback, give compression to the ankle, and are not rigid like the hinged braces therefore making them more comfortable, but do restrict some motion in the sagittal plane.^[17]^

### 2.4. Statistical analysis

The joint position was defined as 3 rotation degree of freedoms (DOFs) during different phases of ankle title. These phases were summarized as follows: dorsiflexion/plantarflexion (DF+/PF–), inversion/eversion (IV+/EV–), internal rotation/external rotation (IR+/ER–). The maximum angles of the ankle complex during tilt were defined as the difference between the maximum and minimum rotation angles in each DOF. The Average velocities of the ankle complex during tilt was defined as the ratio between the maximum angle and time. The final output index of EMGs, which was defined as the average EMG, was the integral average of the EMG amplitude obtained during testing. The results of all groups were converted into the corresponding values with reference to those of the FAI without brace group, which were assigned a value of 100 in all comparisons. Statistical analysis was conducted using SPSS 22.0. The Maximum Angles, average Velocities and average EMG were checked via Kolmogorov–Smirnov tests, and both of them did not meet the normal distribution criterion. Therefore, the data of 2 related samples of FAI without a brace and FAI with a brace were statistically analyzed using a Wilcoxon signed-rank test, and the data of the 2 independent samples were statistically analyzed using Mann–Whitney *U* test. Differences were considered statistically significant when *P* < .05.

## 3. Results

### 3.1. Average ankle position

**Figure 3.**
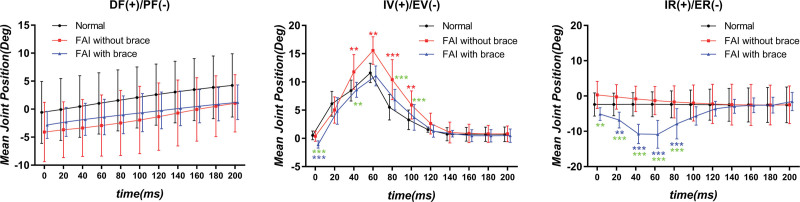
The mean joint position of the ankle complex during tilt (*[red], comparison between FAI without brace and normal; *[blue], comparison between FAI with brace and normal; *[green], comparison between FAI without brace and FAI with brace; ***means *P* < .001, **means *P* < .01, *means *P* < .05); DF+/PF–, dorsiflexion/plantarflexion; IV+/EV–, inversion+/eversion–; IR+/ER–, internal rotation+/external rotation–).

The average ankle position–time curves during tilt are listed in Figure 3. Tilts began at 0 ms.

No significant difference was observed among the 3 groups in terms of DOF of ankle DF/PF. In terms of IV/EV DOF, we found that all 3 groups show the same trend, which started from the neutral position, progressed to gradual IV, and then returned to the neutral position. Furthermore, the FAI without brace group revealed more IV positions at 40 to 100 ms compared with the control group and significantly more IV positions than the FAI with brace group at 0, 40, 80, and 100 ms. The FAI with brace group revealed more EVs than the control group at 0 ms; this finding was the only difference observed between the FAI with brace and control groups at 0 ms. In terms of internal/ER DOF, the FAI with brace group showed more ER positions than the FAI without brace and control groups at 0 to 80 ms. The trend of the curve started from the neutral position, progressed to gradual ER, and then returned to the neutral position. Maximum ER occurred at 60 ms.

### 3.2. Average maximum angles of the ankle complex during tilt

**Figure 4.**
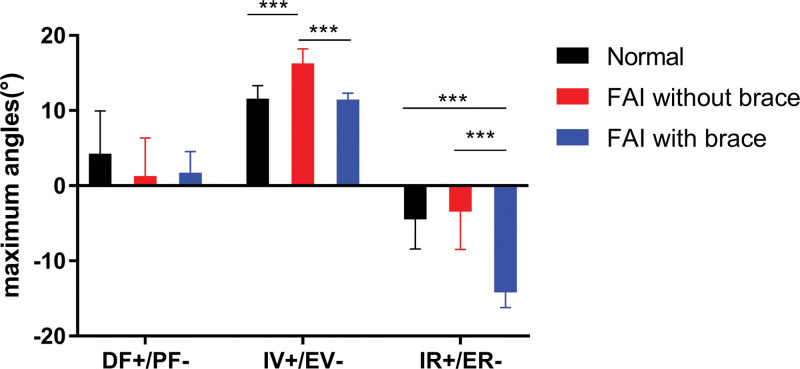
The maximum angles of the ankle complex during tilt (***means *P* < .001, **means *P* < .01, *means *P* < .05); DF+/PF–, dorsiflexion/plantarflexion; IV+/EV–, inversion+/eversion–; IR+/ER–, internal rotation+/external rotation–).

Maximum angles of the ankle complex during tilt are listed in Table 2 and presented in Figure 4. No significant difference was observed among the 3 groups in terms of DOF of ankle DF/PF. In terms of the IV/EV DOF, the FAI without brace group revealed significantly larger angles than the control group (*P* < .001) and significantly more angles than the FAI with brace group (*P* < .001). In terms of internal/ER DOF, the FAI with brace group showed significantly larger angles compared with the FAI without brace (*P* < .001) and the control (*P* < .001) groups.

**Table 2:** The maximum angles of the ankle complex during tilt.

Maximum angles (°)			
	DF+/PF–	IV+/EV–	IR+/ER–
Control	4.26 (5.70)	11.59 (1.71)	–4.47 (3.96)
FAI without brace	1.29 (5.05)	16.27 (1.94)	–3.44 (5.03)
FAI with brace	1.73 (2.82)	11.46 (0.84)	–14.18 (2.02)

DF+/PF– = dorsiflexion/plantarflexion; IV+/EV– = inversion+/eversion–; IR+/ER– = internal rotation+/external rotation–.

### 3.3. Average velocities of the ankle complex during tilt

**Figure 5.**
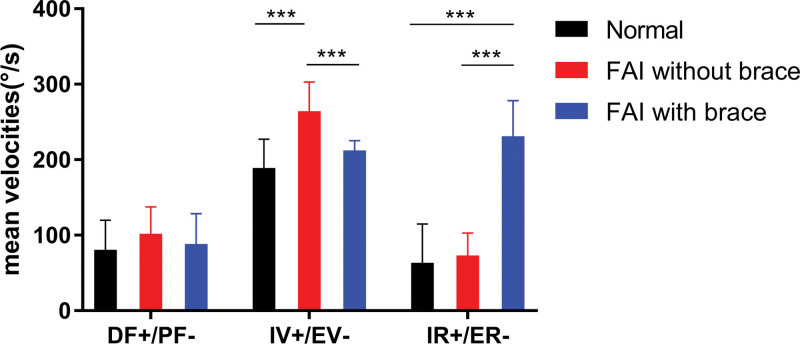
The average velocities of the ankle complex during tilt (***means *P* < .001, **means *P* < .01, *means *P* < .05); DF+/PF–, dorsiflexion/plantarflexion; IV+/EV–, inversion+/eversion–; IR+/ER–, internal rotation+/external rotation–).

Average velocities of the ankle complex during tilt are listed in Table 3 and Figure 5. No significant difference was observed among the 3 groups in terms of DOF of ankle DF/PF. In terms of IV/EV DOF, the FAI without brace group had significantly higher velocities than the control (*P* < .001) and FAI with brace (*P* < .001) groups. In terms of internal/ER DOF, the FAI with brace group showed significantly higher velocities compared with the FAI without brace (*P* < .001) and control (*P* < .001) groups.

**Table 3:** The average velocities of the ankle complex during tilt.

Average velocities (°/s)			
	DF+/PF–	IV+/EV–	IR+/ER–
Control	80.74 (39.24)	189.15 (38.16)	63.55 (51.23)
FAI without brace	101.88 (35.70)	264.06 (38.65)	73.12 (29.80)
FAI with brace	88.01 (40.65)	212.27 (12.89)	231.26 (46.83)

DF+/PF– = dorsiflexion/plantarflexion; IV+/EV– = inversion+/eversion–; IR+/ER– = internal rotation+/external rotation–.

### 3.4. The average EMG of the lower extremity during tilt

The average EMGs of the lower extremity during tilt are listed in Table 4. Compared with the FAI without brace group, the FAI with brace group revealed significantly increased EMG_Prep_ (*P* = .047), EMG_Tilt_ (*P* = .037), and EMG_afterTilt_ (*P* = .004) of the PL. Significant differences were not observed in other muscles.

**Table 4:** The muscular activity of lower extremity during the tilt.

	FAI without brace	FAI with brace
*m. Tibialis anterior*		
EMG_Pre_	100 (0.0)	83.8 (27.8)
EMG_Tilt_	184.3 (0.0)	169.5 (59.1)
EMG_afterTilt_	126.1 (0.0)	112.4 (22.6)
*m. Peroneus longus*		
EMG_Pre_	100 (0.0)	88.3 (13.4)*
EMG_Tilt_	324.8 (27.6)	273.5 (39.3)*
EMG_afterTilt_	212.7 (13.8)	182.3 (19.9)**
*m. Soleus*		
EMG_Pre_	100 (0.0)	93.5 (23.2)
EMG_Tilt_	275.6 (70.6)	285.5 (78.1)
EMG_afterTilt_	189.9 (56.8)	166.9 (32.9)

** means *P* < .01.

* means *P* < .05.

## 4. Discussion

While the ankle brace has demonstrated its efficacy in reducing the recurrence of ankle sprains, particularly among athletes,^[18]^ there remains a gap in understanding its impact on the severity of injuries. Some studies have found that use of lace-up ankle braces reduced the incidence but not the severity of acute ankle injuries in male and female high school basketball athletes both with and without a previous history of an ankle injury.^[19]^ However, another study has discovered that ankle brace can prevent or reduce injuries by affecting proprioception during sudden IV.^[20]^ Therefore, conducting biomechanical research on the role of braces during the instant of a sprain can provide further clarity on the specific mechanisms by which braces affect ankle sprains. However, much of the existing evidence is based on studies that have induced sudden ankle IV in static positions, such as standing or sitting, which may not accurately reflect the functional demands experienced during activities like walking or landing.^[5,6,21,22]^ This limitation underscores the importance of simulating more functional situations to better understand the role of ankle braces in injury prevention. Several studies have attempted to address this gap by simulating ankle sprain under more dynamic conditions, such as walking or landing, conclude that the ankle brace or patient’s mental preparation may reduce some of the muscle activation that occurs during ankle IV.^[7–9]^ However, the interpretation of these results is complicated by the lack of data on synchronous kinematic changes associated with wearing an ankle brace. Only 1 recent study has conducted coupled analysis of the neuromuscular functions and kinematics of the ankle complex.^[23]^ In this work, the researchers induced rapid sudden ankle IV on normal subjects and found that an ankle brace could simultaneously restrict IV and reduce the activity of the PL. It is important to note that the current study focused exclusively on healthy subjects, limiting the generalizability of the findings to patients with FAI. Therefore, our study aimed to fill this knowledge gap by examining the effects of a semirigid ankle brace on kinematic and neuromuscular functions in patients with FAI during induced ankle IV while walking. By extending the scope of research to this population, we hope to gain a more nuanced understanding of how ankle braces may influence injury risk and recovery in individuals with ankle instability.

We studied the kinematics and neuromuscular function of the ankle simultaneously. This current study found some new mechanism of ankle brace during sprain. Ankle brace could effectively reduce the angles and velocities of patients with FAI under rapidly induced ankle IV movement and synchronously reduce PL activity. We also primarily found a compensatory ER mechanism, except when under the direct restriction effect of ankle brace. These findings may provide novel insights into the mechanism of the ankle brace.

When tilt began, the time-related IV angle of the 3 groups rapidly increased and then slowly decreased. We found no difference in the peak angles of the 3 groups. The FAI without brace group had larger maximum IV angles, larger maximum IV velocities, and steeper average ankle position–time curves than the 2 other groups. However, maximum IV angles and velocities significantly decreased after wearing of the ankle brace. These findings are in accordance with previous studies. Lower IV angles result in less ATFL and CFL strain, which indicates that the ankle brace can decrease the strain of lateral ligaments at the instance of sprain.^[24]^ The decrease in ligament strain may contribute to a reduction in the severity of injury when sprain occurs. Besides, significant ER synchronous with IV was observed in the FAI with brace group. A previous biomechanics study showed that compensatory ER of the talocrural joint occurs during extreme ankle IV; this rotation probably occurs against the IV and adjusts the position of the angle. However, the largest tilt angle observed in the current study was 30°, and results showed no IV angles over 20°. Considering that compensatory ER may not occur under our experimental conditions, we did not find ER in the FAI without brace and control groups. Nevertheless, we suppose that the ER occurring in the FAI with brace group was activated in advance by the restriction and proprioception regulation of the ankle brace, thus confirming that injury may be reduced after the brace is worm. Moreover, increases in ER may decrease the strain of ATFL, which may be another protective mechanism of the brace.^[25]^

The sEMG data showed significantly less activation of the PL in the FAI with brace group compared with that in the FAI without brace group. The mechanical support provided by the ankle brace may reduce the demand for muscles when walks or sprain occurs. However, these results contradict previous studies showing increased peroneal stretch-reflex sensitivity^[5,26]^ and motoneuron pool excitability^[27]^ after wearing a brace. Considering that the subjects were tested under sitting or standing conditions in those studies, we feel that our study is more reflective of the actual situation. While our conclusions are similar to those of Barlow,^[28]^ Gehring^[23]^ and Feger.^[29]^ Barlow G showed that ankle bracing resulted in lower precontact amplitude of the peroneus longus comparing with no brace during walking in patients with chronic ankle instability. Gehring induced rapid sudden ankle IV on normal subjects and found that an ankle brace could reduce the activity of the PL. In Feger research, patients with chronic ankle instability demonstrated decreased muscle activity of ankle, knee, and hip musculature during common functional rehabilitative tasks.In the current study, we consider the combined existence of feedforward and feedback regulations when the sEMG of the PL is reduced. On the one hand, the restriction provided by the ankle brace stimulates cutaneous sensation and sends these impulses to the nerve center; thus, regulation starts before the myotatic reflex of the PL, which corresponds to sEMG reductions before tilt. On the other hand, after tilt begins, the nerve center controls the reflex to an appropriate level according to the subject’s needs, which corresponds to sEMG reduction during tilt, due to the weaker myotatic reflex caused by restriction of the ankle brace. We believe that the relatively low activation of the PL during rapidly induced ankle IV could relieve muscle fatigue, which contributes to further injury.^[30]^

Some methodological limitations should be considered when interpreting our results. First, some risks resulting in injury during rapidly induced ankle IV exist. Fortunately, no accident occurred during our experiment. Second, the Vicon Motion System is not as accurate as the 3D–2D fluoroscopy image registration technique used in our previous studies,^[31,32]^ Thus, the kinematics of the talocrural and subtalar joints were not studied separately. Third, considering the large individual differences in sEMG observed, we only conducted self-control before and after patients in the FAI group wore their ankle brace. Finally, accurate comparisons between different groups require a larger sample size.

## 5. Conclusions

Patients with FAI revealed larger IV angles and velocities than normal controls during ankle sprain. Ankle braces can effectively decrease IV angles and velocities during ankle sprain in patients with FAI and simultaneously increase ER angles and velocities. Moreover, braces can decrease the activity of the PL muscle during ankle sprain.

## Author contributions

**Conceptualization:** Gonghao Zhang, Yunhan Ji.

**Data curation:** Chaochao Zha, Shengxuan Cao.

**Formal analysis:** Li Xiong, Guoning Zhang.

**Methodology:** Ping Huang.

**Writing – original draft:** Gonghao Zhang, Chaochao Zha, Shengxuan Cao.

**Writing – review & editing:** Yunhan Ji.
